# Venereal Warts

**Published:** 1882-03

**Authors:** 


### VENEREAL WARTS.

A writer in the *British Medical Journal* has successfully removed these growths by powdering over the surface twice daily with equal parts of burnt alum and tannin. As these growths occur chiefly in situations where mucus or skin surfaces are in contact and moist, this plan suggested itself. In the first case in which he applied it, the warts were easily rubbed off in the course of three or four days, and other cases, have given equally good results.

